# Feasibility Study on Using Dynamic Contrast Enhanced MRI to Assess the Effect of Tyrosine Kinase Inhibitor Therapy within the STAR Trial of Metastatic Renal Cell Cancer

**DOI:** 10.3390/diagnostics11071302

**Published:** 2021-07-20

**Authors:** Jim Zhong, Ebrahim Palkhi, David L. Buckley, Fiona J. Collinson, Christy Ralph, Satinder Jagdev, Naveen S. Vasudev, Jayne Swain, Janet E. Brown, Tze Min Wah

**Affiliations:** 1 Department of Diagnostic and Interventional Radiology, Leeds Teaching Hospitals NHS Trust, Leeds LS9 7TF, UK; jim.zhong@nhs.net (J.Z.); ebrahim.palkhi@nhs.net (E.P.); 2 Biomedical Imaging, School of Medicine, University of Leeds, Leeds LS2 9JT, UK; d.l.buckley@leeds.ac.uk; 3 Department of Medical Oncology, St James’s Institute of Oncology, Leeds LS9 7TF, UK; fiona.collinson@nhs.net (F.J.C.); christy.ralph@nhs.net (C.R.); satinder.jagdev@nhs.net (S.J.); n.vasudev@leeds.ac.uk (N.S.V.); 4 Clinical Trials Research Unit, Leeds Institute of Clinical Trials Research, University of Leeds, Leeds LS9 7TF, UK; j.swain@leeds.ac.uk; 5 Department of Medical Oncology and Metabolism, University of Sheffield, Sheffield S10 2TN, UK; j.e.brown@sheffield.ac.uk

**Keywords:** dynamic contrast-enhanced magnetic resonance imaging (DCE-MRI), tyrosine kinase inhibitor, anti-angiogenic therapy, metastatic renal cell cancer

## Abstract

*Objective*: To identify dynamic contrast-enhanced magnetic resonance imaging (DCE-MRI) parameters predictive of early disease progression in patients with metastatic renal cell cancer (mRCC) treated with anti-angiogenic tyrosine kinase inhibitors (TKI). *Methods*: The study was linked to a phase II/III randomised control trial. Patients underwent DCE-MRI before, at 4- and 10-weeks after initiation of TKI. DCE-MRI parameters at each time-point were derived from a single-compartment tracer kinetic model, following semi-automated tumour segmentation by two independent readers. Primary endpoint was correlation of DCE-MRI parameters with disease progression at 6-months. Receiver operating characteristic (ROC) curve analysis and area under the curve (AUC) values were calculated for parameters associated with disease progression at 6 months. Inter-observer agreement was assessed using the intraclass correlation coefficient (ICC). *Results*: 23 tumours in 14 patients were measurable. Three patients had disease progression at 6 months. The percentage (%) change in perfused tumour volume between baseline and 4-week DCE-MRI (*p* = 0.016), mean transfer constant K^trans^ change (*p* = 0.038), and % change in extracellular volume (*p* = 0.009) between 4- and 10-week MRI, correlated with early disease progression (AUC 0.879 for each parameter). Inter-observer agreement was excellent for perfused tumour volume, K^trans^ and extracellular volume (ICC: 0.928, 0.949, 0.910 respectively). *Conclusions*: Early measurement of DCE-MRI biomarkers of tumour perfusion at 4- and 10-weeks predicts disease progression at 6-months following TKI therapy in mRCC.

## 1. Introduction

Kidney cancer is the third most common genitourinary malignancy, with over 400,000 new diagnoses per year, over 175,000 deaths worldwide annually and a rising incidence [1,2,3]. Metastatic renal cell carcinoma (mRCC) is the most lethal form of this disease, with estimated 5-year survival rates of 11.6% [1,3,4]. Vascular endothelial growth factor receptor (VEGFR)-targeted tyrosine kinase inhibitors (TKIs), including sunitinib and pazopanib, have been effective in treating patients with mRCC by targeting angiogenic pathways but at the expense of significant side-effects [5,6]. Furthermore, it is recognised that not all patients initially respond to TKIs and eventually develop treatment resistance, with median progression-free survival in the order of 8–10 months [5].

Determining whether a patient is continuing to derive benefit from treatment with each cycle can be difficult as traditional imaging assessment criteria, such as Response Evaluation Criteria in Solid Tumours (RECIST), are limited to evaluating size changes that may be slow to occur [7]. Changes in physiology, such as tumour blood flow, precede morphological changes and potentially enables earlier response assessment [8,9]. Currently, there are no validated imaging biomarkers that predict which patients with mRCC will benefit from anti-angiogenic therapy [10]. There are increasing treatment options for such patients and an early indication that they were not responding to treatment would allow switching to alternative therapies earlier.

The STAR trial is a prospective randomised multi-stage phase II/III study of standard continuous TKI vs. a drug-free interval strategy (DFIS) with planned treatment breaks in the first-line treatment of locally advanced/metastatic RCC [11]. As part of the STAR trial, a translational feasibility substudy of the role of dynamic contrast enhanced magnetic resonance imaging (DCE-MRI) in early response assessment was designed to identify predictive biomarkers of response and/or disease progression which would allow early optimisation of treatment schedules and prevent unnecessary toxicity and costs. In light of recent evidence, TKI monotherapy is no longer the first-line treatment for RCC [12] however the assessment of treatment effects remains invaluable.

MRI has advantages for functional imaging including high spatial and contrast resolutions. Previous feasibility studies have shown RCC perfusion can be measured with DCE-MRI before and after targeted intervention and further optimisation of DCE-MRI techniques enables quantification of perfusion [13,14], a technique with clear implications for RCC [15,16,17].

The aim of this study was to investigate if DCE-MRI-based parameters (perfused tumour volume, the transfer constant K^trans^, extracellular volume (ECV) and extracellular mean transit time (MTT)) at 4- and 10-weeks could predict progressive disease (PD) at 6-months after initiation of TKI therapy.

## 2. Materials and Methods

### 2.1. Patient Recruitment and Intervention

Participants in this DCE-MRI translational functional imaging substudy were identified following their recruitment to the STAR trial under full ethical approval (Trial protocol number HTA 09/91/21; Research ethics committee reference number 11/NW/0246 and integrated Research Application System number 75784). All patients were recruited from the single tertiary cancer centre. Study entry criteria required all patients to have pathologically proved clear cell histology, either from nephrectomy specimen or diagnostic biopsy (11). This substudy was an optional part of the STAR trial [11] and participants signed an additional Institutional Review Board approved consent. Patients were required to have measurable disease within the abdomen or pelvis to be eligible for the substudy. For bony metastases, only those with a measurable soft tissue component were included. Participants were required to undergo a baseline contrast-enhanced computed tomography (CECT) and DCE-MRI scan prior to commencement of TKI treatment. The full substudy protocol can be found via the link https://njl-admin.nihr.ac.uk/document/download/2032323 (Last Accessed 10 July 2021). All patients had routine renal function biochemical testing prior to MRI to ensure eGFR was > 30 mL/min before administering contrast. No patients with severe chronic renal impairment were included.

Within the STAR trial, all patients received either pazopanib (800 mg once daily, continuously) or sunitinib (50 mg once daily, days 1 to 28) based on a standard 42-day cycle. Dose modifications were permitted, in line with standard clinical practice.

Participants were randomised prior to starting TKI treatment to either the control arm, continuation of the TKI until evidence of disease progression or toxicity precluded further treatment, or to the experimental/DFIS arm where patients objectively benefiting from treatment temporarily stopped the TKI after a minimum of 4 cycles (a planned treatment break). Participants in the DFIS arm remained off treatment until radiologically confirmed evidence of disease progression, at which point treatment was resumed [11]. Participants in this DCE-MRI sub-study were all taking TKI as per standard practice as the DCE-MRI time points were all before the time that patients took up their allocated arm within the STAR trial (i.e., before participants on the DFIS arm had a planned treatment break). Treatment response at 6-months was assessed by CECT using RECIST version 1.1 criteria [7].

### 2.2. Dynamic Contrast Enhanced Magnetic Resonance Imaging (DCE-MRI)

All DCE-MRI examinations were performed on a Siemens (Erlangen, Germany) 1.5 T system. The examination was performed for up to five target lesions (the largest five) within the abdomen and pelvis identified at baseline (before initiating TKI therapy), 4 weeks (after start of TKI therapy) (±4 days) and 10 weeks (±4 days) following randomisation within the STAR trial.

A dedicated four-element body array coil and integrated spine coil were used for signal reception. The MRI examination included T1- and T2-weighted sequences in the axial, sagittal and coronal planes for morphological imaging. The perfusion assessment with DCE-MRI was performed in an oblique coronal plane to include both kidneys and a section of the descending aorta [14]. The acquired volume was adapted to ensure the five largest abdominal or pelvic lesions (identified on baseline imaging) were included. A pre-contrast T1-weighted 3D fast low angle shot (FLASH) breath-hold sequence (end-expiratory) was performed to establish a signal baseline (6 repeats at 2 s intervals). The DCE-MRI was then performed under gentle breathing for 4 min (120 repeats at 2.4 s intervals). The contrast agent Dotarem (Gd-DOTA Gadoteric acid, Guerbet, France) at a dose of 0.1 mmol/kg body weight was injected into an antecubital vein followed by a 20 mL saline chaser, both at a flow rate of 4 mL/s. The acquisition of the data was triggered at the same time as the contrast agent injection. The scanning range and parameters were copied from the pre-contrast breath hold T1 weighted sequence. The sequence parameters were repetition time 2.38 ms, echo time 1.19 ms, flip angle 19 degrees, field of view 400 × 400 mm, matrix size 128 × 256, slice thickness 10 mm and voxel volume 48.8 mm^3^.

In addition, pre- and post-contrast T1 weighted volume interpolated breath-hold examination (VIBE) sequences were performed for morphological treatment assessment in both axial and coronal planes.

### 2.3. Post DCE-MRI Acquisition Image Analysis

**Figure 1:**
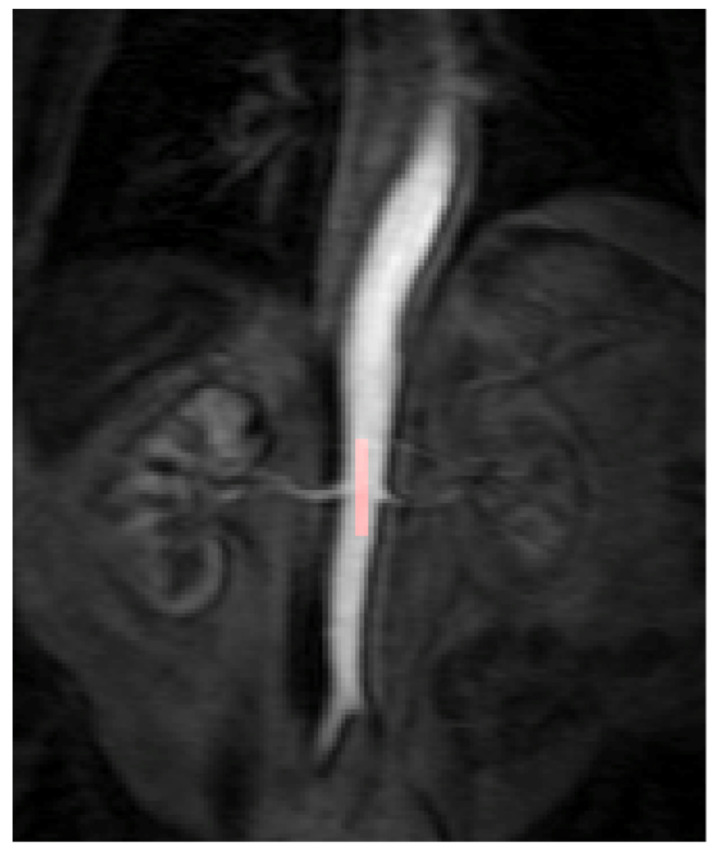
The arterial input function (AIF) region of interest (ROI) was manually drawn inside the aorta at the approximate level of the origin of the vascular pedicles of the kidneys in the dynamic series. The AIF area was optimised further to include only voxels that had a peak relative signal intensity change between the 95th and 100th percentile of the signal change maximum.

All DCE-MRI imaging data were anonymised and post-processed using the software Platform for Research in Medical Imaging Version 0.4 (PMI 0.4) [15]. PMI was used to draw a region of interest (ROI) in the descending aorta and each of the tumours in order to obtain the arterial input function (AIF) and the corresponding tumour perfusion curve. The target tumour lesions identified for each patient within the abdomen and pelvis were segmented by two independent readers (both radiologists) who were blinded to the treatment outcomes and to the other reader’s segmentations. This was repeated for each of the 3 DCE-MRI time points (baseline, 4 weeks and 10 weeks) per patient. To calculate the AIF, a ROI was drawn over the abdominal aorta at the level of the ostia of the renal arteries (Figure 1). For consistency the ROIs were drawn in the phase of the peak contrast enhancement within the abdominal aorta for all patients. The ROI was optimised further to include only voxels that had a peak relative signal intensity change between the 95th and 100th percentile of the signal change maximum. This reproducible thresholding technique was used to reduce the likelihood of inflow or partial voluming effects and has been utilised in previous studies [18].

**Figure 2:**
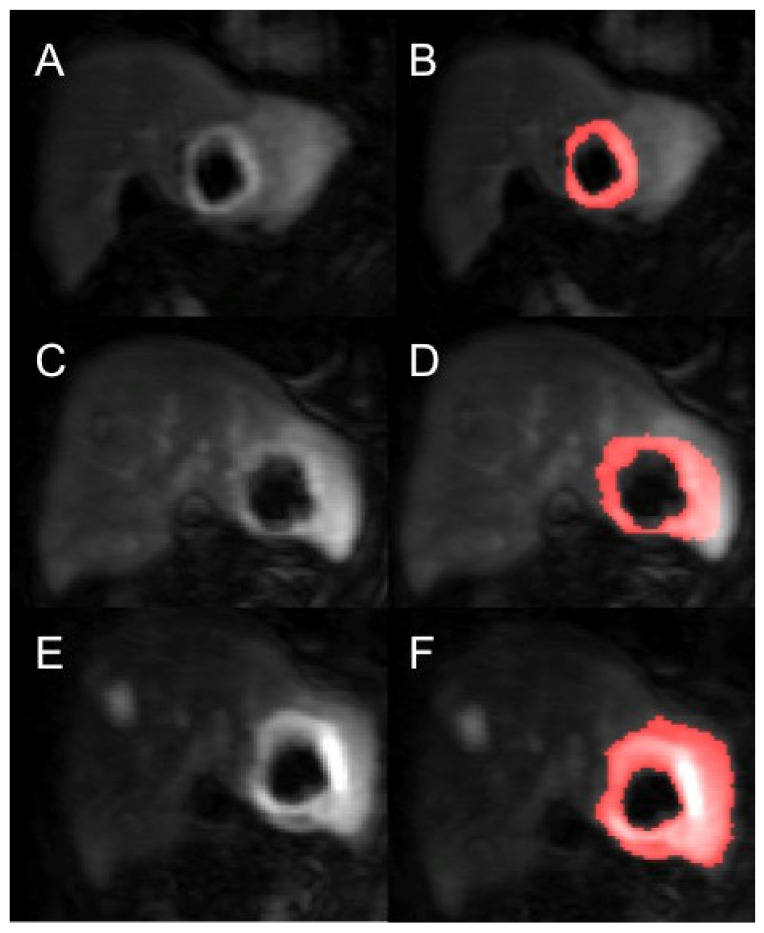
Example of a segmented renal metastasis on DCE-MRI within the left lobe of liver (before and after segmentation with thresholding technique of tumour ROI—the red highlighted area is the tumour ROI segmented on the PMI software) at baseline (**A**,**B**), 4-weeks (**C**,**D**) and 10-weeks (**E**,**F**). This is a case of disease progression at 6 months.

Contrast-agent concentration-time curves were approximated using relative change in signal (compared to baseline) against time [15]. Both readers examined all the MRI datasets (T1 and T2-weighted volumes as well as the post-contrast dynamic data) to help locate the tumours. To assist in identifying the perfused tumour and drawing the perfused tumour ROI, a map of maximum contrast agent concentration was generated. Using the map, tumour ROIs were drawn to encompass the entirety of the perfused tumour, non-enhancing areas and surrounding peri-tumoural tissue. This was done on each image slice that the tumour was present in to generate a 3-dimensional ROI. Using a percentage thresholding technique, the perfused renal tumour volume was more accurately selected to avoid the most peripheral voxels of the lesion to avoid partial volume effects. Using this method, only the perfused tumour was analysed rather than surrounding normal parenchyma or necrotic tissue. This was repeated for every measurable lesion on the DCE-MRI and for all three time points for each patient (Figure 2).

For the calculation of tumour perfusion parameters, a single compartment tracer kinetic model was used [19]. During image quantification and parameter extraction the readers were blinded to patient outcomes. The perfusion parameters extracted were perfused tumour volume, K^trans^ (min^−1^), ECV (mL/100 mL tissue) and their ratio, ECV MTT (s).

### 2.4. Statistical Analysis

The 2-tailed paired *t*-tests were used to analyse the change in the DCE-MRI parameters between the 3 time points (baseline and 4 weeks, 4 weeks and 10 weeks and baseline and 10 weeks). The differences in DCE-MRI parameters between the patients with PD at 6 months and those with no progression were evaluated using an independent samples *t*-test for normally distributed data and the Mann-Whitney U test was used for non-parametric data determined by using a Kolmogorov–Smirnov normality test. For patients with more than one lesion identified on the DCE-MRI, only the largest lesion was selected to analyse the changes to tumour perfusion characteristics over the 3 time points in relation to the primary endpoint as these were least affected by partial volume effect. Receiver operating characteristic (ROC) curve analysis and area under the curve (AUC) values were calculated for parameters that were associated with PD at 6 months. The statistical significance level was set at *p* < 0.05. All statistical tests were performed using SPSS (Version 21.0; IBM Corp., Armonk, New York, NY, USA).

Inter-observer agreement was assessed using the intraclass correlation coefficient (ICC) with ICC values scored as excellent (>0.81), good (0.61–0.80), moderate (0.41–0.60), fair (0.21–0.40), and poor agreement (<0.2).

## 3. Results

**Figure 3:**
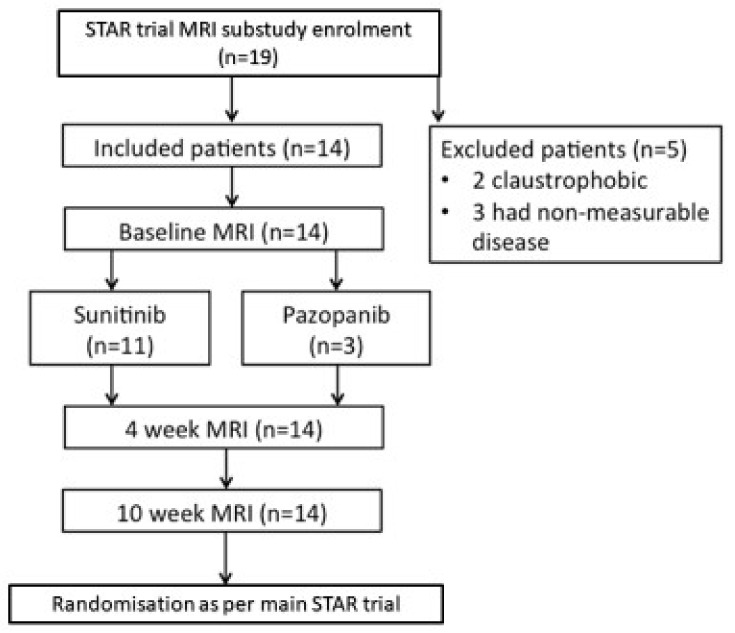
DCE-MRI Study Flowchart.

A total of 19 patients were initially consented and enrolled into this substudy of the STAR trial however 5 patients were excluded due to inability to tolerate the MRI scan due to claustrophobia (*n* = 2) and non-measurable diffuse disease on MRI (*n* = 3). The study flow-chart is shown in Figure 3.

Fourteen patients were included in this substudy. Amongst them, there were 12 male and two female patients. The median age was 64 years (range 52–77). Median Karnofsky performance was 90% (range 80–100). Baseline treatments are presented in Table 1. Three patients had PD at 6 months, 10 had stable disease and one had a partial response.

**Figure 4:**
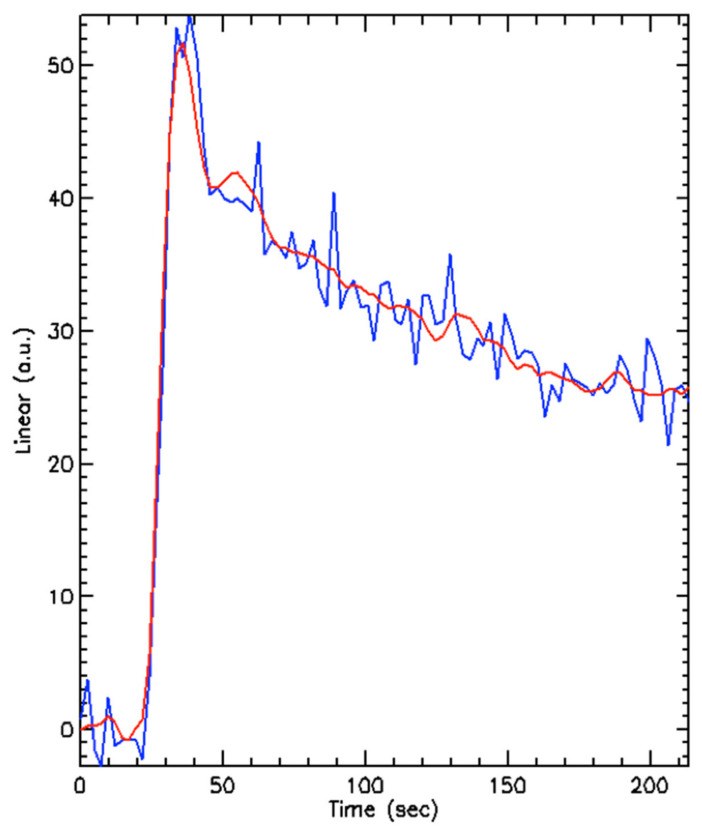
An example of the enhancement curve produced by a segmented tumour (blue line) and a model fit (red line) showing a typical initial peak in uptake of contrast with rapid washout. a.u. = arbitrary units.

A total of 23 separate tumours were measurable. The target lesion sites were: kidney (*n* = 8), nodal (*n* = 6), liver (*n* = 3), pancreas (*n* = 3), stomach (*n* = 1), spleen (*n* = 1) and renal bed (*n* = 1) (Supplementary Materials). The time-intensity curves for each segmented tumour were produced (Figure 4) to which single compartment model fits provided estimates of the perfusion parameters. Only the perfusion parameters of the largest lesion per patient were included in the subsequent analysis below. The perfused tumour volume (cm^3^), K^trans^, ECV (mL/100 mL) and ECV MTT (s) estimates per patient for each tumour at every study time point with percentage changes are shown in Tables S1–S3 in Supplementary Materials.

The median perfused baseline tumour volume was 77.5 cm^3^ (range 2.5–880). The median perfused tumour volume at 4 weeks was 57.7 cm^3^ (range 1.6–600.8) (median percentage change of −48% from baseline, range −92 to +8.6%) (*p* < 0.001). The median perfused tumour volume at 10 weeks was 57.2 cm^3^ (0.2–801.6) (median percentage change of 13% from the 4-week MRI, range −89 to 706%) (*p* = 0.115). The median percentage change from baseline to 10-weeks was −32.8% (range −93 to 83%) (*p* = 0.01).

**Figure 5:**
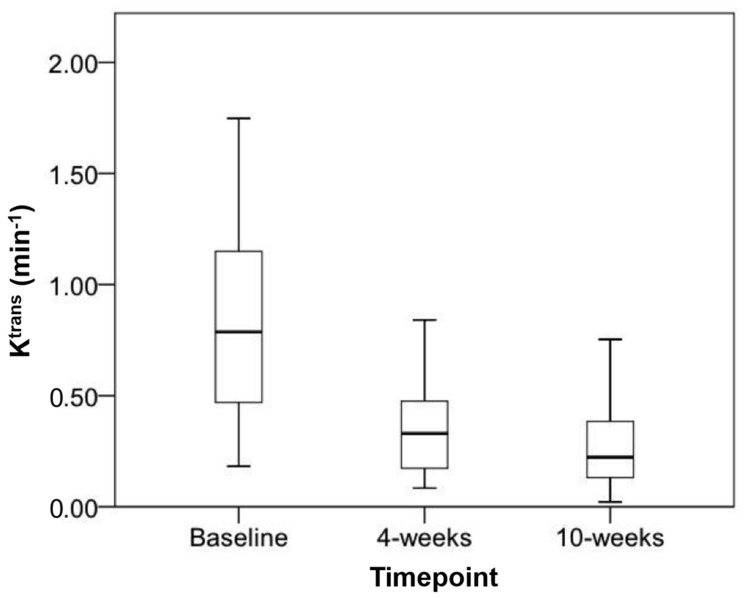
Boxplot of mean K^trans^ at baseline, 4-weeks and 10-weeks with markers representing upper and lower quartiles along with highest and lowest values.

The mean K^trans^ (min^−1^) (±SD) decreased significantly from baseline (0.96 ± 0.63) to 4-weeks (0.37 ± 0.24) (*p* = 0.006) and from baseline to 10-weeks (0.46 ± 0.51) (*p* = 0.033) (Figure 4). The mean K^trans^ change between the 4-weeks and 10-weeks was not significant (*p* = 0.33) (Figure 5). The mean absolute change in K^trans^ between 4- and 10-weeks in the 6-month disease progression group compared to the group without disease progression at 6-months group were +43.9 min^−1^ and −0.4 min^−1^ respectively. This was statistically significant (*p* = 0.038).

**Figure 6:**
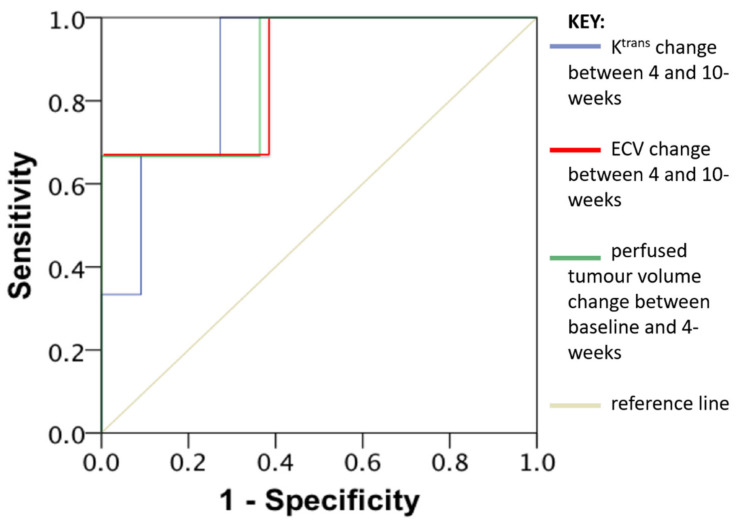
Receiver operating characteristic (ROC) curve analysis and area under the curve (AUC) values of K^trans^ change between 4 and 10-weeks (Blue line), Extracellular volume change (ECV) between 4 and 10-weeks (Red line) and perfused tumour volume change between baseline and 4-weeks (Green line). AUC 0.879 for all three of these parameters individually.

The following parameters were associated with early disease progression at 6 months: percentage change in perfused tumour volume between baseline and 4-weeks (*p* = 0.016), K^trans^ change between 4- and 10-weeks (*p* = 0.038) and percentage change in ECV between 4- and 10-weeks (*p* = 0.009). ROC curve analysis found the AUC values to be 0.879 for all three of these parameters individually (ROC curve shown in Figure 6).

### Interobserver Agreement

The inter-observer agreement was excellent for perfused tumour volume, K^trans^ and ECV across all segmented lesions with semi-automated ROI placement. Perfused tumour volume (ICC: 0.928; 95% confidence interval [CI]: 0.869, 0.959). K^trans^ (ICC: 0.949; 95% confidence interval [CI]: 0.918, 0.969). ECV (ICC: 0.910; 95% confidence interval [CI]: 0.800, 0.961).

## 4. Discussion

RCC tumour biology is characterised by angiogenesis and hypervascularity due to increased expression of VEGF resulting in endothelial proliferation and neo-vessel formation [17,20,21]. This makes RCC an optimal target for measuring tumour perfusion and highlights the clinical relevance of evaluating efficacy of anti-angiogenic TKIs, which inhibit VEGF receptor signalling, resulting in reduced microvascular density [17,20,22]. Changes in tumour vessel density have been shown to correlate with response and resistance to anti-angiogenic therapy [23].

The measurement of K^trans^, a DCE-MRI derived quantitative marker of microvascular function and surrogate for tumour blood flow [19], potentially provides a non-invasive imaging biomarker to quantify the decrease in microvascular function. The main findings of this prospective imaging biomarker substudy are that absolute and relative changes in DCE-MRI derived quantitative biomarkers (perfused tumour volume, K^trans^ and ECV) at the 4- and 10-weeks MRI scan following TKI treatment were correlated with early disease progression at 6-months.

To date, this is the first clinical study to use longitudinal serial assessments to detect changes in quantitative DCE-MRI biomarkers following sunitinib or pazopanib treatment in mRCC. The inclusion of patients treated with either TKI is based on the similar mechanism of action and efficacy [6,11]. Previous studies investigating the effect of anti-angiogenic treatment on DCE-MRI biomarkers in mRCC are summarised in Table 2. A lack of standardised DCE-MRI parameters and measurements taken at different time points precludes direct comparison between the studies.

The reduction in K^trans^ at 4- and 10-weeks relative to baseline after starting anti-angiogenic therapy are in line with previous studies [24,25]. The decrease in perfused tumour volume at 4-weeks may be attributed to early changes in microvasculature induced by the anti-angiogenic therapy, which occur as early as three days after treatment starting [26,27]. Prior studies have demonstrated a decrease in K^trans^ when the tumour responds to the TKI treatment [28,29]. These findings support the concept that inhibition of angiogenesis may be a key independent predictor of outcome [17,24,25]. Baseline pre-treatment K^trans^ has previously been shown to be predictive of those responding to anti-angiogenic treatment and significantly associated with progression free survival [17,24,25,26,30,31,32]. Although we did not find baseline K^trans^ to be predictive of early disease progression at 6 months, this is likely due to our small sample size with few patients (*n* = 3) progressing at the 6-month interval.

The cases where K^trans^ increased between 4- and 10-weeks were correlated with disease progression at 6 months despite all these patients still having stable disease by RECIST criteria at 10-weeks. This finding is supported by pre-clinical studies which suggest that changes in blood flow, including rising K^trans^, precedes tumour growth [22,33]. This again highlights the potential capability of K^trans^ to be an early biomarker of treatment efficacy and importantly before any tumour size change is observed. Between 4- and 10-weeks, continued anti-angiogenic treatment would be expected to further reduce K^trans^ therefore an increase instead may suggest early signs of treatment resistance and/or disease relapse as observed in all three patents with early disease progression.

A similar study by Sweis et al. where patients with mRCC were treated with 28-day cycles of pazopanib, with MRI scans at 8-, 16- and 24-weeks, found the decrease in K^trans^ relative to baseline was observed at all time points [25]. There was an observed trend towards K^trans^ recovery by 24-weeks, which preceded the median progression free survival by 8-weeks and therefore potentially an earlier marker of disease progression [25].

The varying responses of metastatic tumours within the same patient as reflected by the different degrees of change in the perfusion parameters between lesions in the same patient (Supplementary Materials) also highlights the complex inter-tumoural heterogeneity in mRCC known to contribute to the heterogeneous clinical outcomes observed in clinical trials [20]. Functional imaging may allow for further quantification of tumour heterogeneity to help understand tumour progression further in patients undergoing anti-angiogenic therapy before morphological changes in size occur [34].

The choice of tumour ROI selection was informed by previous research on DCE-MRI which has shown advantages of using whole-tumour ROIs compared to smaller ROIs with higher inter-observer correlation for DCE perfusion and permeability parameters [35]. This method has been previously demonstrated in other cancer types [36]. The high ICC confirms that this semi-automated method of measuring perfused tumour volume on DCE-MRI images is reproducible. Across institutions, the issue of inter-algorithm variability when evaluating DCE-MRI results in dramatically different parameters and is a major obstacle to implementing DCE-MRI into practice [37,38,39].

The strength of this prospective study is that it is linked to a phase II/III trial where longitudinal data is still being collected for the survivors. Limitations include the small sample size and inclusion of multiple lesions from a variety of organs sites, which have variable baseline perfusion however to account for these differences we used both absolute and percentage changes between each DCE-MRI time point. A few tumours were difficult to delineate due involvement of other well-perfused organs or structures such as the spleen, liver, or abdominal arteries. Although two different types of targeted therapy (sunitinib or pazopanib) were used, previous DCE-MRI studies show both anti-angiogenic agents result in similar trends in reduction of K^trans^ with similar efficacy [6,24,25].

## 5. Conclusions

This study supports further investigation of DCE-MRI derived biomarkers of tumour perfusion (perfused tumour volume, K^trans^ and extracellular volume) as potential surrogate biomarkers to predict disease progression following TKI therapy in metastatic RCC. We have demonstrated that this approach is feasible and of interest; further larger studies are required to test its wider clinical application.

## Figures and Tables

**Table 1:** Patient treatment characteristics with targeted therapy regimen.

Patient	Prior Nephrectomy	Sites of Disease/Index Lesions	Targeted Therapy	Progressive Disease at 6 Months
1	Yes	Nodal	Sunitinib	No
2	Yes	Spleen/Stomach	Sunitinib	No
3	Yes	Nodal	Sunitinib	No
4	Yes	Liver (2)	Sunitinib	Yes
5	Yes	Nodal	Sunitinib	Yes
6	No	Kidney	Sunitinib	No
7	No	Kidney	Pazopanib	No
8	No	Kidney	Sunitinib	No
9	No	Kidney	Pazopanib	No
10	Yes	Nephrectomy bed/Nodal	Sunitinib	No
11	Yes	Nodal (2)	Sunitinib	No
12	Yes	Kidney (2)/Liver/Pancreas (2)	Pazopanib	No
13	No	Kidney/Pancreas	Sunitinib	Yes
14	No	Kidney	Sunitinib	No

() = number of tumours if more than one.

**Table 2:** Previous published clinical studies using DCE-MRI to assess response to anti-angiogenic therapy in renal cancer.

Authors [Reference]	Year	Number of Patients Included in Analysis	Anti-Angiogenic Therapy
Flaherty et al. [17]	2008	15	Sorafenib
Hahn et al. [24]	2008	44	Sorafenib
Sweis et al. [25]	2017	17	Pazopanib
Hudson et al. [26]	2018	34	Sunitinib
Desar et al. [27]	2011	10	Sunitinib

## Data Availability

The data used to support the findings of this study are available from the corresponding author upon request.

## Supplementary Materials

The following are available online at https://www.mdpi.com/article/10.3390/diagnostics11071302/s1. Supplementary Tables (Table S1 Baseline and follow-up perfused tumour volume (cm^3^) estimates per measured tumour target lesion for each patient with percentage changes, Table S2 Baseline and follow-up K^trans^ (/min) estimates per measured tumor target lesion for each patient with percentage changes, Table S3 Baseline and follow-up extra-cellular volume (ECV) (mL/100 mL) estimates per measured tumour target lesion for each patient with percentage changes and Table S4 Baseline and follow-up ECV mean transit time (MTT) (s) estimates per measured tumour target lesion for each patient with percentage changes.).

## References

[1] Bray F., Ferlay J., Soerjomataram I., Siegel R.L., Torre L.A., Jemal A. (2018). Global cancer statistics 2018: GLOBOCAN estimates of inci-dence and mortality worldwide for 36 cancers in 185 countries. CA Cancer J. Clin..

[2] Siegel R.L., Miller K.D., Jemal A. (2018). Cancer statistics, 2018. CA Cancer J. Clin..

[3] Saad A., Gad M.M., Al-Husseini M.J., Ruhban I.A., Sonbol M., Ho T.H. (2019). Trends in renal-cell carcinoma incidence and mortality in the United States in the last 2 decades: A SEER-based study. Clin. Genitourin. Cancer.

[4] National Cancer Institute Surveillance, Epidemiology and End Results (SEER) Program, 2017. https://seer.cancer.gov/.

[5] Motzer R.J., Hutson T.E., Tomczak P., Michaelson D., Bukowski R.M., Rixe O., Oudard S., Negrier S., Szczylik C., Kim S.T. (2007). Sunitinib versus interferon alfa in metastatic renal-cell carcinoma. N. Engl. J. Med..

[6] Motzer R.J., Hutson T., Cella D., Reeves J., Hawkins R., Guo J., Nathan P., Staehler M., De Souza P., Merchan J.R. (2013). Pazopanib versus sunitinib in metastatic renal-cell carcinoma. N. Engl. J. Med..

[7] Eisenhauer E.A., Therasse P., Bogaerts J., Schwartz L.H., Sargent D., Ford R., Dancey J., Arbuck J., Gwyther S., Mooney M. (2009). New response evaluation criteria in solid tumours: Revised RECIST guideline (version 1.1). Eur. J. Cancer.

[8] Wasser K., Klein S.K., Fink C., Junkermann H., Sinn H.-P., Zuna I., Knopp M.V., Delorme S. (2003). Evaluation of neoadjuvant chemotherapeutic response of breast cancer using dynamic MRI with high temporal resolution. Eur. Radiol..

[9] Mankoff D.A., Dunnwald L.K., Gralow J.R., Ellis G.K., Schubert E.K., Tseng J., Lawton T.J., Linden H.M., Livingston R.B. (2003). Changes in blood flow and metabolism in locally advanced breast cancer treated with neoadjuvant chemotherapy. J. Nucl. Med..

[10] Fournier L.S., Oudard S., Thiam R., Trinquart L., Banu E., Médioni J., Balvay D., Chatellier G., Frija G., Cuenod C.A. (2010). Metastatic renal carcinoma: Evaluation of antiangiogenic therapy with dynamic contrast-enhanced CT. Radiology.

[11] Collinson F.J., Gregory W.M., McCabe C., Howard H., Lowe C., Potrata D.B., Tubeuf S., Hanlon P., McParland L., Wah T. (2012). The STAR trial protocol: A randomised multi-stage phase II/III study of sunitinib comparing temporary cessation with allowing continuation, at the time of maximal radiological response, in the first-line treatment of locally advanced/metastatic renal cancer. BMC Cancer.

[12] Choueiri T., Powles T., Burotto M., Bourlon M., Zurawski B., Juárez V.O., Hsieh J., Basso U., Shah A., Suarez C. (2020). 696O_PR Nivolumab + cabozantinib vs. sunitinib in first-line treatment for advanced renal cell carcinoma: First results from the randomized phase III CheckMate 9ER trial. Ann. Oncol..

[13] Chapman S., Wah T.M., Sourbron S., Buckley D. (2013). The effects of cryoablation on renal cell carcinoma perfusion and glomerular filtration rate measured using dynamic contrast-enhanced MRI: A feasibility study. Clin. Radiol..

[14] Wah T., Sourbron S., Wilson D., Magee D., Gregory W., Selby P., Buckley D.L. (2018). Renal cell carcinoma perfusion before and after radiofrequency ablation measured with dynamic contrast enhanced MRI: A pilot study. Diagnostics.

[15] Notohamiprodjo M., Sourbron S., Staehler M., Michaely H.J., Attenberger U.I., Schmidt G.P., Boehm H., Horng A., Glaser C., Stief C. (2010). Measuring perfusion and permeability in renal cell carcinoma with dynamic contrast-enhanced MRI: A pilot study. J. Magn. Reson. Imaging.

[16] Rosen M.A., Schnall M.D. (2007). Dynamic contrast-enhanced magnetic resonance imaging for assessing tumor vascularity and vascular effects of targeted therapies in renal cell carcinoma. Clin. Cancer Res..

[17] Flaherty K.T., Rosen M.A., Heitjan D.F., Gallagher M.L., Schwartz B., Schnall M.D., O’Dwyer P.J. (2008). Pilot study of DCE-MRI to predict progression-free survival with sorafenib therapy in renal cell carcinoma. Cancer Biol. Ther..

[18] Georgiou L., Wilson D.J., Sharma N., Perren T.J., Buckley D.L. (2018). A functional form for a representative individual arterial input function measured from a population using high temporal resolution DCE MRI. Magn. Reson. Med..

[19] Sourbron S.P., Buckley D. (2011). On the scope and interpretation of the Tofts models for DCE-MRI. Magn. Reson. Med..

[20] Hsieh J.J., Purdue M.P., Signoretti S., Swanton C., Albiges L., Schmidinger M., Heng D.Y., Larkin J., Ficarra V. (2017). Renal cell carcinoma. Nat. Rev. Dis. Prim..

[21] Ng C.S., Wang X., Faria S.C., Lin E., Charnsangavej C., Tannir N.M. (2010). Perfusion CT in patients with metastatic renal cell carcinoma treated with interferon. Am. J. Roentgenol..

[22] Bhatt R.S., Wang X., Zhang L., Collins M., Signoretti S., Alsop D., Goldberg S.N., Atkins M.B., Mier J.W. (2010). Renal cancer resistance to antiangiogenic therapy is delayed by restoration of angiostatic signaling. Mol. Cancer Ther..

[23] Vasudev N.S., Goh V., Juttla J.K., Thompson V.L., Larkin J.M.G., Gore M., Nathan P.D., Reynolds A.R. (2013). Changes in tumour vessel density upon treatment with anti-angiogenic agents: Relationship with response and resistance to therapy. Br. J. Cancer.

[24] Hahn O.M., Yang C., Medved M., Karczmar G., Kistner E., Karrison T., Manchen E., Mitchell M., Ratain M.J., Stadler W.M. (2008). Dynamic contrast-enhanced magnetic resonance imaging pharmacodynamic biomarker study of sorafenib in metastatic renal carcinoma. J. Clin. Oncol..

[25] Sweis R., Medved M., Towey S., Karczmar G.S., Oto A., Szmulewitz R.Z., O’Donnell P.H., Fishkin P., Karrison T., Stadler W.M. (2017). Dynamic contrast-enhanced magnetic resonance imaging as a pharmacodynamic biomarker for pazopanib in metastatic renal carcinoma. Clin. Genitourin. Cancer.

[26] Hudson J.M., Bailey C., Atri M., Stanisz G., Milot L., Williams R., Kiss A., Burns P.N., Bjarnason G.A. (2018). The prognostic and predictive value of vascular response parameters measured by dynamic contrast-enhanced-CT, -MRI and -US in patients with metastatic renal cell carcinoma receiving sunitinib. Eur. Radiol..

[27] Desar I., Ter Voert E., Hambrock T., Van Asten J., Van Spronsen D., Mulders M., Heerschap A., Van Der Graaf W., Van Laarhoven H., Van Herpen C. (2011). Functional MRI techniques demonstrate early vascular changes in renal cell cancer patients treated with sunitinib: A pilot study. Cancer Imaging.

[28] O’Connor J., Jackson A., Parker G., Roberts C., Jayson G. (2012). Dynamic contrast-enhanced MRI in clinical trials of antivascular therapies. Nat. Rev. Clin. Oncol..

[29] Akisik M.F., Sandrasegaran K., Bu G., Lin C., Hutchins G.D., Chiorean E.G. (2010). Pancreatic cancer: Utility of dynamic contrast-enhanced MR Imaging in assessment of antiangiogenic therapy. Radiology.

[30] Piludu F., Marzi S., Pace A., Villani V., Fabi A., Carapella C.M., Terrenato I., Antenucci A., Vidiri A. (2015). Early biomarkers from dynamic contrast-enhanced magnetic resonance imaging to predict the response to antiangiogenic therapy in high-grade gliomas. Neuroradiology.

[31] Hsu C.Y., Shen Y.C., Yu C.W., Hsu C., Hu F.C., Hsu C.H., Chen B.-B., Wei S.-Y., Cheng A.-L., Shih T.T.-F. (2011). Dynamic contrast-enhanced magnetic resonance imaging biomarkers predict survival and response in hepatocellular carcinoma patients treated with sorafenib and metronomic tegafur/uracil. J. Hepatol..

[32] Saito K., Ledsam J., Sugimoto K., Sourbron S., Araki Y., Tokuuye K. (2018). DCE-MRI for early prediction of response in hepatocellular carcinoma after TACE and sorafenib therapy: A pilot study. J. Belg. Soc. Radiol..

[33] Huang D., Ding Y., Zhou M., Rini B.I., Petillo D., Qian C.-N., Kahnoski R., Futreal P.A., Furge K.A., Teh B.T. (2010). Interleukin-8 mediates resistance to antiangiogenic agent sunitinib in renal cell carcinoma. Cancer Res..

[34] Fournier L., Bellucci A., Vano Y., Bouaboula M., Thibault C., Elaidi R., Oudard S., Cuenod C. (2017). Imaging response of antiangiogenic and immune-oncology drugs in metastatic renal cell carcinoma (mRCC): Current status and future challenges. Kidney Cancer.

[35] Braunagel M., Radler E., Ingrisch M., Staehler M., Schmid-Tannwald C., Rist C., Nikolaou K., Reiser M., Notohamiprodjo M. (2015). Dynamic contrast-enhanced magnetic resonance imaging measurements in renal cell carcinoma: Effect of region of interest size and positioning on interobserver and intraobserver variability. Invest. Radiol..

[36] Goh V., Halligan S., Gharpuray A., Wellsted D., Sundin J., Bartram C.I. (2008). Quantitative assessment of colorectal cancer tumor vascular parameters by using perfusion CT: Influence of tumor region of interest. Radiology.

[37] Beuzit L., Eliat P.-A., Brun V., Ferré J., Gandon Y., Bannier E., Saint-Jalmes H. (2015). Dynamic contrast-enhanced MRI: Study of inter-software accuracy and reproducibility using simulated and clinical data. J. Magn. Reson. Imaging.

[38] Heye T., Davenport M.S., Horvath J.J., Feuerlein S., Breault S.R., Bashir M.R., Merkle E.M., Boll D.T. (2013). Reproducibility of dynamic contrast-enhanced MR Imaging. Part I. Perfusion characteristics in the female pelvis by using multiple computer-aided diagnosis perfusion analysis solutions. Radiology.

[39] Huang W., Li X., Chen Y., Li X., Chang M.-C., Oborski M.J., Malyarenko D.I., Muzi M., Jajamovich G.H., Fedorov A. (2014). Variations of dynamic contrast-enhanced magnetic resonance imaging in evaluation of breast cancer therapy response: A multicenter data analysis challenge. Transl. Oncol..

