# Supplementary material for: Feasibility Study on Using Dynamic Contrast Enhanced MRI to Assess the Effect of Tyrosine Kinase Inhibitor Therapy within the STAR Trial of Metastatic Renal Cell Cancer
Source: Diagnostics (Basel). 2021 Jul 20;11(7):1302. doi: 10.3390/diagnostics11071302 (PMC8306403; doi:10.3390/diagnostics11071302)
Supplement: Supplementary file 1 [file diagnostics-11-01302-s001.zip › diagnostics-1244214-supplementary.pdf]

Supplementary Information

Table S1. Baseline and follow-up perfused tumour volume (cm^3) estimates per measured tumour target lesion for each patient with percentage changes.

Patient	Target Lesion Site	Baseline MRI	4-week Follow-up MRI	% Change from Last Scan	10-week Follow-up MRI	% Change from Last Scan
1	Node	2.5	1.6	−34.2%	0.2	−89.1%
2	1) Spleen	160.5	88.5	−44.8%	83.7	−5.5%
	2) Gastric	437.0	107.9	−75.3%	801.6	643.0%
3	Node	3.2	1.6	−51.1%	3.7	131.8%
4	1) Right lobe liver	21.8	25.4	16.7%	47.1	85.2%
	2) Left lobe liver	67.8	73.6	8.6%	84.1	14.2%
5	Node	221.5	117.7	−46.8%	212.4	80.4%
6	Left Kidney	9.9	4.8	−51.1%	3.3	−31.2%
7	Right Kidney	688.7	354.5	−48.5%	453.5	27.9%
8	Left Kidney	214.9	94.1	−56.2%	64.9	−31.1%
9	Right Kidney	72.2	41.8	−42.1%	46.7	11.7%
10	1) Nephrectomy Surgical Bed	72.1	41.2	−42.8%	49.5	20.0%
	2) Node	30.9	19.4	−37.3%	8.7	−55.2%
11	1) Node	45.2	18.1	−60.1%	9.8	−45.6%
	2) Node	82.8	34.9	−57.9%	27.2	−22.0%
12	1) Left Liver	14.2	7.6	−46.5%	19.9	162.5%
	2) Left Kidney (anterior)	31.3	4.0	−87.2%	29.0	626.3%
	3) Left Kidney (lower)	21.9	9.0	−58.9%	13.9	54.8%
	4) Pancreas body	24.9	7.1	−71.6%	14.4	102.7%
	5) Pancreas tail	21.5	1.7	−92.3%	13.3	705.8%
13	1) Left Kidney	426.1	361.7	−15.1%	296.4	−18.1%
	2) Pancreas	36.2	19.8	−45.1%	14.1	−29.1%
14	Right Kidney	880.4	600.8	−31.8%	233.7	−61.1%

Table S2. Baseline and follow-up K^{trans} (/min) estimates per measured tumor target lesion for each patient with percentage changes.

Patient	Target Lesion Site	Baseline MRI	4-week Follow-up MRI	% Change from Last Scan	10-week Follow-up MRI	% Change from Last Scan
1	Node	0.79	0.08	−90%	0.07	−13%
2	1) Spleen	0.36	0.02	−94%	0.13	550%
	2) Gastric	0.18	0.12	−33%	0.13	8%
3	Node	2.52	0.16	−94%	0.15	−6%
4	1) Right lobe liver	0.95	0.57	−40%	1.36	139%
	2) Left lobe liver	0.97	0.79	−19%	1.76	123%
5	Node	1.15	0.25	−78%	0.59	136%
6	Left Kidney	0.47	0.43	−9%	0.75	74%
7	Right Kidney	0.58	0.39	−33%	0.27	−31%
8	Left Kidney	1.75	0.3	−83%	0.24	−20%
9	Right Kidney	1.19	0.84	−29%	1.36	62%
10	1) Nephrectomy Surgical Bed	0.43	0.48	12%	0.31	−35%
	2) Node	0.43	0.56	30%	0.31	−45%
11	1) Node	1.04	0.13	−88%	0.18	38%
	2) Node	0.47	0.36	−23%	0.02	−94%
12	1) Left Liver	0.5	0.21	−58%	0.17	−19%
	2) Left Kidney (anterior)	0.31	0.59	90%	0.45	−24%
	3) Left Kidney (lower)	0.72	1.03	43%	1.07	4%
	4) Pancreas body	1.56	0.39	−75%	1.25	221%
	5) Pancreas tail	1.82	0.11	−94%	1.03	836%
13	1) Left Kidney	0.49	0.2	−59%	0.21	5%
	2) Pancreas	1.09	0.39	−64%	0.62	59%
14	Right Kidney	0.95	0.17	−82%	0.13	−24%

Table S3. Baseline and follow-up extra-cellular volume (ECV) (mL/100 mL) estimates per measured tumour target lesion for each patient with percentage changes.

Patient	Target Lesion Site	Baseline MRI	4-week Follow-up MRI	% Change from Last Scan	10-week Follow-up MRI	% Change from Last Scan
1	Node	33	4	−87%	7	62%
2	1) Spleen	13	7	−47%	5	−21%
	2) Gastric	12	19	64%	7	−63%
3	Node	31	7	−77%	6	−12%
4	1) Right lobe liver	35	17	−50%	42	140%
	2) Left lobe liver	28	16	−42%	32	96%
5	Node	24	11	−53%	28	157%
6	Left Kidney	10	18	88%	16	−11%
7	Right Kidney	16	16	1%	14	−15%
8	Left Kidney	29	14	−50%	17	19%
9	Right Kidney	27	24	−13%	30	25%
10	1) Nephrectomy Surgical Bed	19	20	3%	15	−25%
	2) Node	19	14	−24%	6	−55%
11	1) Node	17	8	−53%	11	36%
	2) Node	10	16	52%	4	−76%
12	1) Left Liver	14	23	68%	7	−68%
	2) Left Kidney (anterior)	18	35	98%	21	−40%
	3) Left Kidney (lower)	24	74	208%	39	−48%
	4) Pancreas body	22	24	11%	21	−15%
	5) Pancreas tail	23	5	−78%	18	238%
13	1) Left Kidney	16	18	18%	19	3%
	2) Pancreas	12	16	32%	18	10%
14	Right Kidney	33	13	−62%	13	5%

Table S4. Baseline and follow-up ECV mean transit time (MTT) (s) estimates per measured tumour target lesion for each patient with percentage changes.

Patient	Target Lesion Site	Baseline MRI	4-week Follow-up MRI	% Change from Last Scan	10-week Follow-up MRI	% Change from Last Scan
1	Node	26	30	18%	63	110%
2	1) Spleen	21	174	713%	24	−86%
	2) Gastric	38	97	152%	32	−67%
3	Node	7	28	272%	26	−6%
4	1) Right lobe liver	22	18	−17%	18	1%
	2) Left lobe liver	18	13	−28%	11	−12%
5	Node	12	26	114%	29	9%
6	Left Kidney	12	25	105%	13	−49%
7	Right Kidney	16	24	49%	30	25%
8	Left Kidney	10	29	192%	43	48%
9	Right Kidney	14	17	24%	10	−43%
10	1) Nephrectomy Surgical Bed	27	25	−6%	28	14%
	2) Node	26	15	−41%	12	−20%
11	1) Node	10	37	281%	37	−1%
	2) Node	13	26	97%	102	290%
12	1) Left Liver	16	64	298%	25	−61%
	2) Left Kidney (anterior)	34	36	4%	28	−22%
	3) Left Kidney (lower)	20	43	114%	22	−50%
	4) Pancreas body	8	37	344%	10	−73%
	5) Pancreas tail	8	28	257%	10	−63%
13	1) Left Kidney	19	56	192%	55	−2%
	2) Pancreas	7	25	265%	18	−29%
14	Right Kidney	21	44	111%	60	38%
